# Enzyme Inhibitor May Offer Dual Protection against Brain Disease

**DOI:** 10.1371/journal.pbio.1001270

**Published:** 2012-02-21

**Authors:** Charles Q. Choi

**Affiliations:** Freelance Science Writer, New York, New York, United States of America

## Abstract

Genetic analysis in budding yeast and in cultured human astrocytes reveals that specific histone deacetylase complexes accelerate expansion mutations in DNA triplet repeats.

**Figure pbio-1001270-g001:**
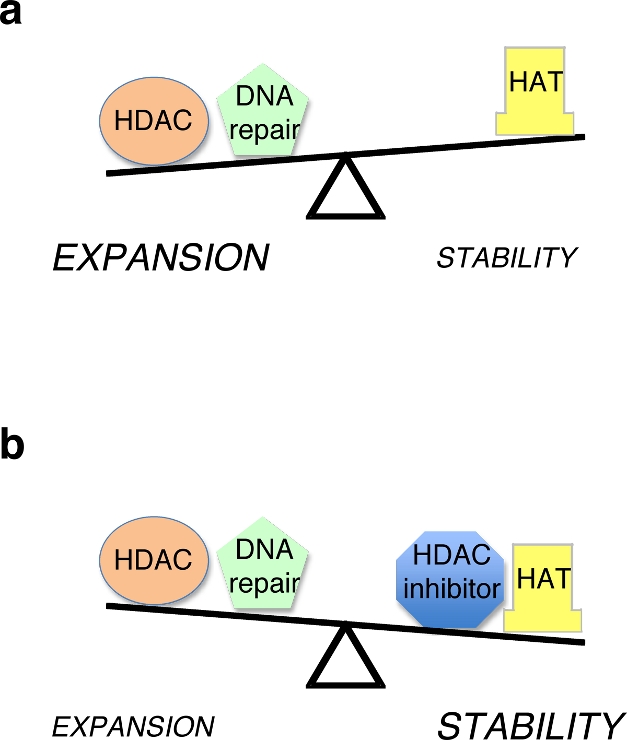
Combined actions of HDACs and DNA repair outweigh HATs, resulting in expansions (a). But perturbing this situation, for example, adding an HDAC inhibitor (b), changes the outcome (stabilizing trinucleotide repeats).

[Fig pbio-1001270-g001]Chromosomes are extraordinarily long, wiggly strands, making up more than two meters of DNA in each human cell, and given the 50 trillion or so cells in the human body, we have enough DNA in each of us to go from here to the sun and back more than 300 times. To wrangle chromosomes into nuclei only ten to 20 microns wide, these molecules are wound tightly around proteins known as histones, compacting them by a factor of 10,000 during cell division.

By controlling access to genetic material, histones are more than just spools, but caretakers as well. Now, Robert Lahue and his colleagues find enzymes that tamper with these guardians can warp the DNA wrapped around them, triggering mutations that are the cause of at least 17 inherited neurological human disorders, including Huntington's disease. There is a silver lining to this discovery: interfering with these enzymes could help treat these diseases in high risk individuals, and some drugs that do just that are already under investigation.

The mutations in question are trinucleotide repeat expansions, where three-nucleotide-long pieces of gene code such as cytosine-adenine-guanine (CAG) get repeatedly inserted into genes. Once these repeats pile up or “expand” past a certain threshold number within genes—say, 30 to 40 uninterrupted repeats in humans—genetic activity can get disrupted, with potentially tragic results.

To find out the mysterious root of these mutations, Lahue and his colleagues started with yeast that already possessed stretches of 20 cytosine-thymine-guanine (CTG) repeats. The more such repeats they accumulate, the more resistant they prove to the toxin canavanine. The researchers dosed yeast cells with mutagens, causing 9,000 random mutations in approximately half of yeast's non-essential genes. Treatment with canavanine then revealed 11 mutant genes that caused persistent vulnerability to the toxin, suggesting they might normally help generate repeats.

Three of these genes were linked with enzymes known as histone deacetylases (HDACs), which remove acetyl groups from histones. Knocking out any one of these genes reduced trinucleotide repeat expansion rates by 50 to 90 percent, confirming their normal ability to help mutate DNA.

To see what might happen in human cells, the scientists exposed lab-grown astrocytes—human nervous system cells that are among the targets of Huntington's disease—to an HDAC inhibitor known as 4b. This suppressed trinucleotide repeat expansion rates by about 75 percent. Using RNA interference to knock down the enzyme HDAC3 in these cells had much the same effect. On the other hand, knocking down enzymes known as histone acetyltransferases that add acetyl groups to histones, the opposite of HDACs, increased expansion frequency.

Lahue and his team's experiments suggest that although there are at least 14 different types of HDACs in human cells, only one or a few seem to affect trinucleotide repeat expansions, such as HDAC3. These might not only generate the inherited cause of Huntington's and other diseases—the trinucleotide repeats—but might further exacerbate these problems during patients' lifetimes by causing more repeats. Lahue said future work will focus on why HDAC3 specifically causes expansions.

The researchers suggest HDACs can inadvertently promote expansions by prolonging the lifetimes of proteins normally involved in DNA repair. Experiments with yeast mutants revealed the DNA-cleaving nuclease Sae2, which recent studies suggest is stabilized by histone deacetylation, appears to be a target of HDACs, promoting expansions by working together with the nuclease Mre11.

The HDAC inhibitor the researchers used, 4b, is already being tested as a treatment in Huntington's disease. The protein huntingtin apparently binds to Argonaute proteins, which are responsible for micro-RNA-mediated gene silencing; HDAC inhibitors affect gene transcription, potentially alleviating the abnormal gene silencing triggered by the mutant huntingtin of Huntington's disease. Intriguingly, it now turns out 4b might not only treat the effects of the disease, but also suppresses the mutations that contribute to the disorder. Lahue hopes to work with other labs to see if blocking HDAC3 in mice helps relieve trinucleotide repeat expansions in the brain. He adds that specific HDAC3 inhibitors might be developed as mutation-blocking therapies for additional human triplet repeat expansion diseases.

**Debacker K, Frizzell A, Gleeson O, Kirkham-McCarthy L, Mertz T, et al. (2012) Histone Deacetylase Complexes Promote Trinucleotide Repeat Expansions. doi:10.1371/journal.pbio.1001257**

